# Promising, but Not Completely Conclusive—The Effect of l-Theanine on Cognitive Performance Based on the Systematic Review and Meta-Analysis of Randomized Placebo-Controlled Clinical Trials

**DOI:** 10.3390/jcm14217710

**Published:** 2025-10-30

**Authors:** Rebeka Olga Mátyus, Zsóka Szikora, Diána Bodó, Bettina Vargáné Szabó, Éva Csupor, Dezső Csupor, Barbara Tóth

**Affiliations:** 1 Faculty of Pharmacy, Institute of Clinical Pharmacy, University of Szeged, 6725 Szeged, Hungary or matyus.rebeka@szte.hu (R.O.M.); szikora.zsoka@szte.hu (Z.S.); vargane.szabo.bettina@szte.hu (B.V.S.); 2 Department of Pediatrics and Pediatric Health Center, Albert Szent-Györgyi Health Centre, 6725 Szeged, Hungary

**Keywords:** l-theanine, green tea, *Camellia sinensis*, cognition, cognitive function, meta-analysis

## Abstract

**Background:** Green tea (*Camellia sinensis*) has been consumed for centuries, and its beneficial effects on human health have been studied in recent decades. l-theanine, an active ingredient in green tea, has been used to improve cognition and mood. Although the effects of l-theanine on cognition have been investigated in clinical trials that have reported various results, these studies have not yet been critically evaluated in meta-analyses. **Objectives:** Our objective was to systematically evaluate the efficacy of l-theanine on cognitive functions compared to a placebo, in a meta-analysis based on randomized controlled trials (RCTs). **Methods:** PubMed, the Cochrane Central Register of Controlled Trials, Embase and Web of Science were searched for relevant studies until 31 July 2024 and registered in PROSPERO (registration number: CRD42024575122). Placebo-controlled clinical trials investigating the efficacy of l-theanine in healthy adults were included. Conference abstracts, study protocols and reports of non-RCTs were excluded. For risk of bias assessment, the Cochrane Risk of Bias Tool (version 2.0) was used. A random effects model was applied to conduct the meta-analysis. Mean differences (MD) with 95% confidence intervals (CIs) were calculated. **Results:** Based on the included five RCTs involving 148 healthy adults, l-theanine had a dose-dependent effect on cognitive function based on rapid visual information processing and recognition visual reaction time (MD: −15.20 ms; 95%-CI [−28.99; −1.41]). The effects of l-theanine were non-significant on reaction time to a simple stimulus (MD: −0.46 ms; 95% [CI: −15.65; 14.73]) and in the Stroop test (MD: −37.38 ms; 95%-CI [−86.39; 11.62]). **Conclusions:** The beneficial effects of l-theanine on cognitive performance could not be confirmed by all test methods. The contradictory results could be explained by the fact that l-theanine only affects certain cognitive domains, but also by the low number of trials and the heterogeneity of the test preparations. Further trials using standardized products with larger sample sizes are required for the accurate assessment of efficacy.

## 1. Introduction

Green tea (*Camellia sinensis*) is an evergreen plant found mainly in the tropical and temperate regions of Asia, especially in China, Srí Lanka, Japan and India, and it has been consumed for centuries as food and for its beneficial effects on human health [1,2,3]. The chemical composition of green tea leaves is well studied [3]. The main constituents of the leaves are polyphenols, but other components such as methylxanthine derivatives (caffeine, theobromine, theophylline), organic acids, flavonoids, free amino acids (such as l-theanine) and several flavor components are also present in fresh leaves [3]. Among other purposes, green tea has also been used to improve cognition and mood. Based on preclinical and clinical evidence, caffeine and l-theanine are the two main constituents contributing to this effect [4]. Caffeine is a stimulant compound belonging to the group of methylxanthines and is found in several plants, including *Coffea arabica* (coffee), *Camellia sinensis* and *Paullinia cupana* (guarana) [3,5].

After oral administration, caffeine is rapidly and completely absorbed from the gastrointestinal tract, reaching maximum plasma concentrations about 1 h after ingestion. The structure of caffeine is adequately hydrophobic to pass through all biological membranes and distribute throughout the body [6]. Caffeine affects a number of organ systems, including the central nervous system, the cardiovascular system, neuroinflammatory, neuromodulatory and neuroprotective processes and the respiratory system [6,7]. Its mechanism of action is characterized by the blockade of adenosine _A1_ and _A2_ receptors [5,6,7,8], and, in higher doses, the inhibition of the phosphodiesterase enzyme and calcium mobilization from intracellular stores in skeletal and cardiac muscle are also observed [6]. Caffeine acts as a stimulant in the central nervous system and increases alertness, reduces fatigue, increases work capacity, improves sustained visual selective attention and target-specific attention and increases behavioral performance in terms of accuracy and reaction speed [5,6,7]. In the cardiovascular system, caffeine exerts rapid positive inotropic and chronotropic effects following oral administration, leading to an increase in heart rate and conductance [5]. The effects of caffeine on the respiratory system include an increased sensitivity of medullary centers to the stimulatory actions of CO_2_ [6].

**Figure 1:**
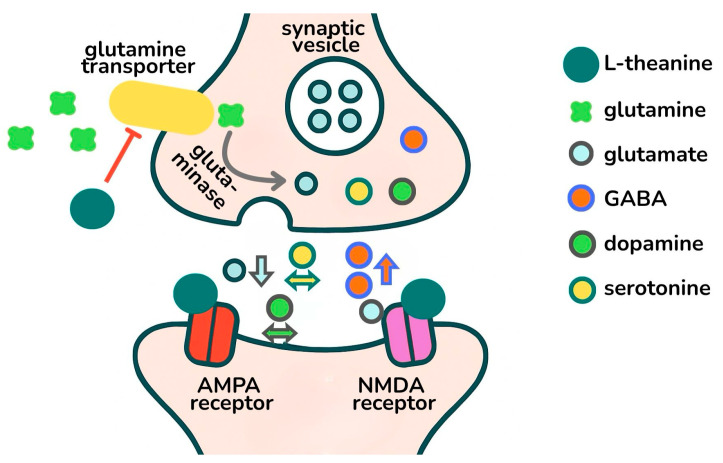
Schematic representation of mechanisms of action of l-theanine in the central nervous system.

In recent years, another constituent of green tea has drawn the interest of researchers. l-theanine is a naturally occurring amino acid found in *Camellia sinensis* that makes up almost 50% of the free amino acids in green tea. l-theanine was first isolated from green tea in 1949, but, apart from green tea, can be found in other plants of the *Camellia* genus, and it has been identified in the non-edible mushroom *Xerocomus badius* [9]. The amount of l-theanine in green tea accounts for 1–3% of the dry weight of tea leaves. This amount varies depending on factors such as the geographical origin of the tea, the time of harvest and the production method. Since its first isolation, l-theanine has been attributed several beneficial effects; therefore, it is not surprising that l-theanine is also marketed in purified form as a food supplement [10].

l-theanine is a water-soluble, non-proteinogenic amino acid with a molecular structure similar to that of glutamic acid [2,4,11,12,13]. l-theanine can cross the blood–brain barrier in 30 min after ingestion in humans, and the maximum plasma concentrations of the component occur 30–120 min after administration and are completely cleared from the plasma in 24 h [12,14,15]. l-theanine has several beneficial effects, including the improvement of mood and cognition and reductions in stress and anxiety-like symptoms [4]. The possible mechanisms of action of l-theanine in the central nervous system involve several targets. Among these, l-theanine inhibits glutamate reuptake and confers a neuroprotective effect by binding to gamma-aminobutyric acid (GABA_A_) receptors [16]. l-theanine acts as a weak antagonist of glutamate on the AMPA and modulates the NMDA receptors. Moreover, it inhibits the glutamine transporter, thereby inhibiting the formation of glutamate from glutamine. l-theanine increases the release of GABA, whereas its effect on dopamine and serotonine levels varies depending on the area of the brain; see Figure 1. [17]. In a systematic review published in 2019 by Williams et al., l-theanine in its pure form at a daily dose of 200–400 mg reduced stress and anxiety in people under acute stress [4]. The effect of theanine on cognitive functions can probably be explained in part by its anxiety- and stress-reducing effects.

As life expectancy increases in aging societies, a growing number of people will be affected by cognitive impairment. Therefore, there is a constant need for safer and more effective therapies that can reverse or slow down cognitive impairment. Based on the above-mentioned studies, it is reasonable to assume that l-theanine might offer a safe and effective way to improve cognitive function. Therefore, our aim was to evaluate the effects of l-theanine compared to a placebo on cognitive function by performing a meta-analysis of randomized, placebo-controlled clinical trials.

## 2. Materials and Methods

### 2.1. Design

This meta-analysis was designed, conducted and reported in accordance with the PRISMA 2020 (Preferred Reporting Items for Systematic Reviews and Meta-Analyses) guidelines, and it was registered in the International Prospective Register of Systematic Reviews (PROSPERO) on 31 July 2024 (registration number: CRD42024575122).

The following PICOS (patients/population, intervention, comparison, outcome, study design) format was applied: P: healthy adults (over 18 years of age), regardless of their tea or caffeine consumption habits; I: known doses of l-theanine given *per os*; C: placebo; O: change in cognitive function based on different scales and visual tests; and S: placebo-controlled randomized clinical trials.

### 2.2. Information Sources and Search Strategy

Literature searches were carried out through the databases Embase, PubMed, Cochrane Central Register of Controlled Trials and Web of Science until 7 December 2024 using the following search strategy: ((*Camellia sinensis*) OR (green tea) OR (l-theanine)) AND (cognitive function).

No language, publication date or publication status restrictions were applied. The reference lists of all identified articles were inspected.

### 2.3. Eligibility Criteria and Study Selection

All randomized, placebo-controlled clinical studies that evaluated the efficacy of l-theanine on cognitive functions in healthy, adult patients were included. Abstracts, case series, case reports, rapid communications, study protocols and articles not reporting numerical data on efficacy were excluded. For reference management, Zotero 7.0 software was used. After removing duplicates, the remaining records were screened for eligibility based on the titles and abstracts. The eligibility of the full texts of the resulting records was independently evaluated by two reviewers (R.M., Z.S.). In cases of disagreement between reviewers, a third reviewer (D.C.) was consulted.

### 2.4. Data Extraction and Synthesis of the Results

Data was collected according to PRISMA guidelines. The two reviewers (R.M., Z.S.) independently selected the characteristics and results of the studies. In cases of disagreement, a third reviewer (B.T.) helped to reach a consensus. The following data items were extracted from the included papers: study design, characteristics of the patient population and sample size, intervention details, type of comparator(s), outcome measures and overall results. Simple reaction time, simple visual reaction time, Stroop test results, rapid visual information processing and recognition visual reaction time values were extracted as outcome measures. Studies were grouped according to outcome measures as defined in the protocol. Study characteristics were tabulated (e.g., population, intervention, comparator, outcomes and study design) and compared against the pre-specified synthesis groups. Only studies that reported the required outcome measures in compatible formats were included in quantitative synthesis. In cases of missing or incomplete data, study authors were contacted.

### 2.5. Risk of Bias Analysis

The risk of bias was evaluated by two of the authors (R.M., Z.S.) using the Cochrane Risk of Bias Tool (version 2.0). Any differences in the evaluation were resolved by consensus. If the risk of bias was low in all domains, the overall risk of bias in each trial was considered low; if the risk of bias was high in at least one domain, the overall risk of bias in each trial was considered high. In any other context, the risk of bias was considered to show some concern.

### 2.6. Statistical Analysis

Statistical analysis was performed using the RStudio software (version 2024.9.0.375) with the meta and metafor packages. Mean differences (MDs) with 95% confidence intervals (CIs) were calculated. A random effects model was applied to conduct the meta-analysis. In this method, the aim is to estimate the mean of a distribution of effects. Study weights are based on the within-study variance plus a constant (τ^2^, the between-study variance). The inclusion of τ^2^ reduces the disparity between study weights, meaning that larger studies have less dominance, while smaller studies gain relatively more influence [18]. Forest plots were used to visually display effect sizes and confidence intervals for each study and pooled results. Data visualization was performed using R metafor package (version 2024.9.0.375). We synthesized the results narratively when it was not possible to include a study in the meta-analysis. Heterogeneity was examined with the Higgins’ I^2^ indicator, which represents the percentage of total variation across studies. According to the Cochrane Handbook, I^2^ can indicate low (0–40%), moderate (30–60%), substantial (50–90%) or considerable heterogeneity (75–100%) [19].

### 2.7. Quality of Evidence

The GRADEpro GDT (Guideline Development Tool) tool was used to evaluate the quality of evidence. Two authors (B.T. and R.M.) independently performed the grading of the level of evidence. Each outcome was rated according to the following: risk of bias, inconsistency, indirectness, imprecision, publication bias, presence of a large effect, dose-dependent response and plausible confounders (“not serious”, “serious” or “very serious”). The final certainty of the evidence was categorized as “very low”, “low”, “moderate” or “high” [20].

## 3. Results

### 3.1. Study Selection

**Figure 2:**
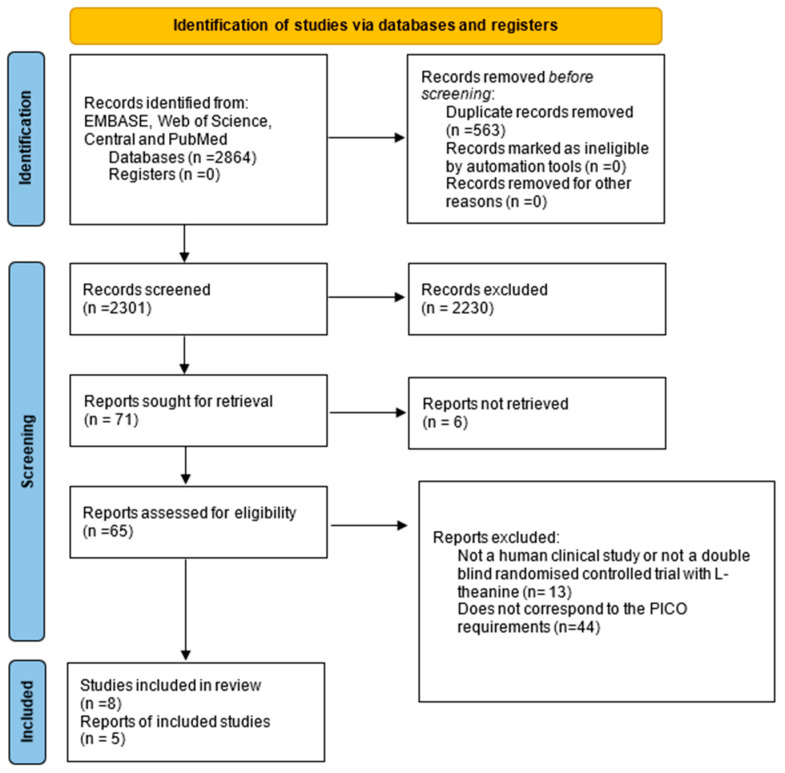
PRISMA 2020 flow diagram for identification of relevant studies.

A literature search was conducted using the search terms “l-theanine” and “cognitive function” in the Embase, PubMed, Cochrane Central Register of Controlled Trials and Web of Science databases. After the removal of duplicates, 2301 potentially relevant reports were collected (see Table S1 in Supplementary Material for excluded studies). Eligible RCTs were selected according to the PRISMA flowchart shown in Figure 2. After screening the titles and abstracts, 71 publications were retrieved for full-text screening, of which 63 articles were also excluded (Supplementary Material Table S1). Of the remaining eight articles, five were eligible for further statistical analysis and three were included in the qualitative analysis.

#### Studies Included in the Quantitative Analysis

A total of five placebo-controlled randomized trials were included in the quantitative analysis (see Table 1). In the included studies, different methods were used to assess the effects of l-theanine on cognitive function. One of these tests was the simple reaction time test, in which participants had to respond as quickly as possible to a simple visual stimulus by pressing a button. Reaction times were measured in milliseconds. Results of simple reaction time from four studies involving a total of 126 participants were included in the quantitative analysis [21,22,23,24].

The so-called Stroop test was applied in three studies involving 96 participants to measure the effects of l-theanine on cognitive function. The Stroop test is a procedure in which a series of color names appear on the screen in succession in different colors. Participants must use a response pad with color codes to select the color that matches the color of the letters in the word. The words that appear are either “congruent” (the color name and the color of the letters are the same) or “incongruent” (the color name and the color of the letters are different) and appear in random order. Participants must respond as quickly and accurately as possible. The timed task was evaluated based on the reaction time and the interference reaction time (the difference between the reaction time to congruent and incongruent stimuli). In this test, the reaction time to congruent and incongruent stimuli were measured in milliseconds [21,23,25]. When calculating incongruent data, we specified the differences compared to congruent data.

The third endpoint was the rapid visual information processing test/recognition visual reaction time test. A total of four studies involving 125 participants were included in the statistical analysis of the time required to process visual stimuli (RVIP/RVRT/continuous performance) [21,22,23,24].

### 3.2. Study Characteristics

The trials included in the statistical analysis were completed in four different countries [Japan (n = 1), the UK (n = 1), Turkey (n = 1), Sri Lanka (n = 2)], from 2008 to 2023. All were randomized, placebo-controlled, double-blind studies. The drug investigated in all cases was l-theanine. The main characteristics of the included studies are presented in Table 1, Table 2, Table 3 and Table 4. Sample sizes ranged from 20 to 50 participants. Doses of l-theanine ranged from 50 mg to approximately 500 mg.

### 3.3. Outcomes

#### 3.3.1. Effects of l-Theanine on Reaction Time to Simple Stimulus

In the analyzed articles [21,22,23,24], the reaction time required for processing a simple stimulus was measured in milliseconds. Dodd et al. (2015) evaluated the effect of 50 mg l-theanine on the simple reaction time in a double-blind, placebo-controlled, counterbalanced, crossover study of 24 healthy participants [21]. The effects were recorded approximately forty minutes after the administration. The 24 subjects were divided into two groups based on whether they were habitual caffeine consumers or not. Twelve people were in each of the two groups. Participants had to respond as quickly as they could when they saw an upward pointing arrow appear on a screen by pressing the space bar. The reaction time was measured in milliseconds [21].

In the study of Kahathuduwa et al. (2016), 200 mg of l-theanine was administered to 20 healthy patients in a placebo-controlled, five-way crossover trial [22]. In the simple visual reaction time test, the participant had to press a reaction time button as quickly as possible in response to a randomly timed white flash appearing in a blue background on the screen approximately 50 min post-dose [22]. The researchers observed that there was no significant time effect (F_(1,19)_ = 0.679, *p* = 0.42, η^2^ = 0.035) or time × treatment interaction (F_(4,76)_ = 0.137, *p* = 0.903, η^2^ = 0.007) in the simple visual reaction time result; therefore, it can be concluded that l-theanine did not cause a significant change in the simple visual reaction time [22].

A double-blind, randomized, placebo-controlled parallel group study by Baba et al. aimed to assess the efficacy of a single dose of 100.6 mg of l-theanine on cognitive function using the Cognitrax test in 50 healthy patients [23]. In the study, 24 people were in the placebo group and 26 people in the l-theanine group. The data for the simple reaction time test were taken from the first part of the Stroop test, in which the participants were asked to perform a task approximately 60 min after the ingestion of the drug, in which they had to press a key as soon as they saw the character written in black letters on the screen. The researchers found that, in the single-dose study, the simple reaction times were significantly lower in the theanine group than in the placebo group [23].

Dassanayake (2023) conducted a double-blind, placebo-controlled, counterbalanced, four-way crossover study involving 32 healthy young adults [24]. The researchers compared the effects of three doses of l-theanine (100, 200 and 400 mg) to placebo (distilled water) on three attentional tasks. The results from before and 50 min after administration were compared. In each of the reported outcomes, three different forest plots were generated to examine the effects of the different doses. In the simple reaction time task, participants had the target displayed at a predictable location. They measured the reaction time for each task [24].

**Figure 3:**
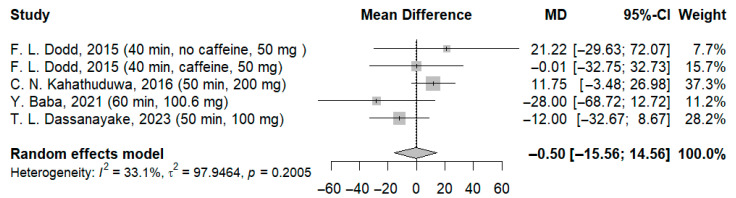
Forest plot of simple reaction time I [21,22,23,24].

**Figure 4:**
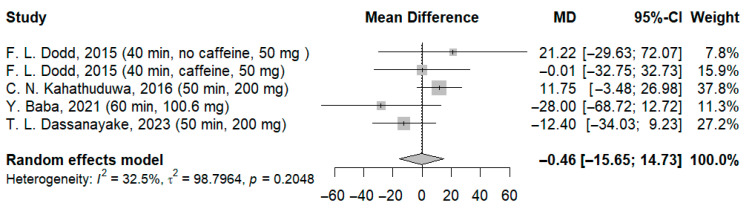
Forest plot of simple reaction time II [21,22,23,24].

In our meta-analysis, data from 126 participants were analyzed in the simple reaction time test, and three forest plots were created, analyzing separately the effects of the different doses of l-theanine reported by Dassanayake et al. When patients receiving 100 mg (Figure 3) and 200 mg (Figure 4) of l-theanine from the aforementioned study were included in the analysis, the results were non-significant. The mean difference was −0.50 ms (95% CI: −15.56; 14.56; I^2^ = 33.1%; *p* = 0.2005) for the former and −0.46 ms (95% CI: −15.65; 14.73; I^2^ = 32.5%; *p* = 0.2048) for the latter.

**Figure 5:**
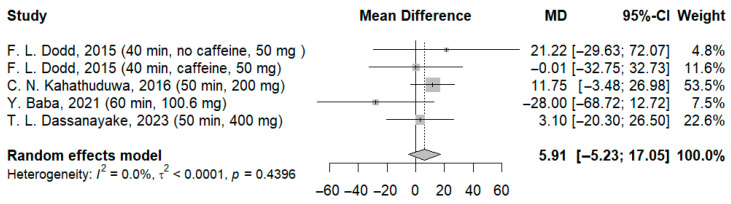
Forest plot of simple reaction time III [21,22,23,24].

When participants who received the highest dose of l-theanine (400 mg) were included in the analysis, a non-significant deterioration was observed compared to the placebo group (MD: 5.91 ms; 95%-CI [−5.23; 17.05]; I^2^ = 0.0%; *p* = 0.4396) (Figure 5).

Currently, there is insufficient scientific literature to explain the deterioration in cognitive function. Further studies are needed to analyze the dose–response effects of l-theanine. To conclude, the acute administration of l-theanine at doses ranging from 50 to 400 mg does not influence the cognitive function of healthy adults based on the simple reaction time results.

#### 3.3.2. Effects of l-Theanine on Stroop Test

The efficacy of l-theanine based on the Stroop test was assessed including data from three studies [21,23,25]. Twelve habitual caffeine users and twelve non-usual caffeine users were involved to the study of Dodd et al. (2015) [21]. Each participant was given 50 mg of l-theanine and placebo in a counterbalanced order across separate visits. Approximately 60 min after the ingestion of the preparation, the participants’ performances on the Stroop test were monitored [21].

The Stroop test was also applied in the study of Baba et al. (2021) [23]. In this double-blind, randomized, placebo-controlled parallel group study, 26 subjects participated in the l-theanine group (100.6 mg) and 24 in the placebo group. The participants performed the Stroop test 60 min after administration. From this study, we analyzed responses both to congruent and incongruent stimuli. In this study, the letters of words such as “red”, “yellow”, “blue” and “green” were displayed in colors red, yellow, blue and green. During the congruent phase, participants were asked to press a button when the meaning of the word matched its color. During the incongruent phase, participants were asked to press a button when the meaning of the word did not match its color [23].

In the double-blind, randomized, controlled crossover study of Yilmaz et al. (2023), twenty-two elite national curling athletes participated [25]. Participants in the group consumed 6 mg/kg of l-theanine, whereas the placebo contained 400 mg of maltodextrin. Participants performed the Stroop test approximately 65 min after consuming l-theanine or the placebo [25]. Participants were instructed to press the “←” or “→” arrow key on the keyboard with their right index and ring fingers. The task in this study consisted of three blocks of 30 neutral, 30 congruent and 30 incongruent stimuli, but we only included the reaction times of responses to congruent and incongruent stimuli in the analysis [25].

**Figure 6:**
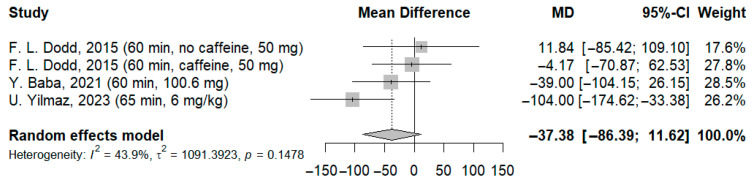
Forest plot of the Stroop test results for congruent stimuli [21,23,25].

A total of three studies involving 96 participants were analyzed based on the results of the Stroop test. The meta-analysis revealed that the reaction time to congruent stimuli did not change significantly compared to the control group (MD: −37.38 ms; 95%-CI [−86.39; 11.62]; I^2^ = 43.9%; *p* = 0.1478) (Figure 6).

**Figure 7:**
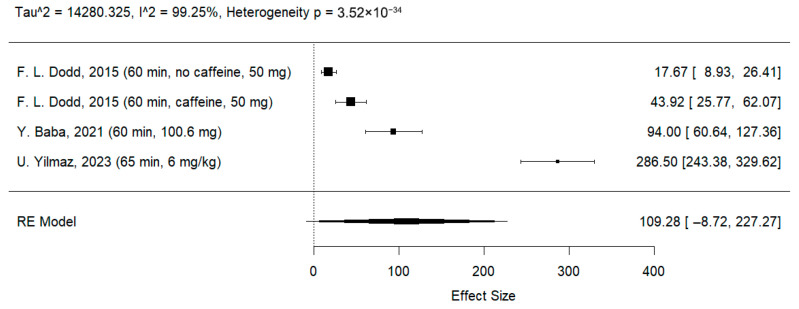
Forest plot of the Stroop test results for incongruent stimuli [21,23,25].

The reaction time to incongruent stimuli increased in the l-theanine group, but this difference was not significant (MD: 109. 28 ms; 95% CI [−8.72; 227.27]; I^2^ = 99.25%; *p* < 0.001) (Figure 7).

#### 3.3.3. Effects of l-Theanine on Time Required to Process Visual Stimuli (RVIP—Rapid Visual Information Processing/RVRT—Recognition Visual Reaction Time/Continuous Performance)

In this part of the meta-analysis, data from four studies were analyzed [21,22,23,24]. In the study of Dodd et al. (2015), 12 habitual caffeine users and 12 non-usual caffeine users were included [21]. They were given 50 mg of l-theanine and placebo in a counterbalanced order across separate visits. The rapid information processing task was performed 50 min after the consumption of the test preparation. The participant had to watch a series of numbers continuously appearing on the monitor screen and look for three consecutive odd or three consecutive even digits. The digits appeared in a random order, and the participants pressed the space bar as quickly as possible when they noticed the target sequence of digits. The task was evaluated based on the average reaction time for correct detections and the number of false alarms [21].

In the study by Kahathuduwa et al. (2016), 200 mg of l-theanine was administered to 20 healthy patients in a placebo-controlled, five-way crossover trial [22]. The test was conducted 55 min after the consumption of the product. Participants were asked to react as quickly as possible to a white flash appearing on the screen and press the reaction time button, while ignoring a red flash appearing with the same probability, which was a distracting stimulus. The reaction time was measured in milliseconds [22].

In the double-blind, randomized placebo-controlled parallel group study conducted by Baba et al. (2021), 26 subjects participated in the l-theanine group and 24 in the placebo group [23]. The test required participants to consume 100.6 mg of l-theanine or a placebo. In the study, participants completed the Cognitrax test, which measures cognitive function, approximately 70 min after consuming the supplement. For the meta-analysis, we used data from one of the Cognitrax subtests, the continuous performance task. In this part of the test, letters of the alphabet appeared in a random order on the screen. Participants were asked to press the button only when the letter “B” appeared. The reaction time for correct answers was measured in milliseconds [23].

Dassanayake (2023) conducted a double-blind, placebo-controlled, counterbalanced, four-way crossover study involving 32 healthy young adults [24]. The researchers compared the effects of three doses of l-theanine (100, 200 and 400 mg) to a placebo (distilled water) on a rapid visual information test. The test was conducted 60 min after the consumption of the preparation. Subjects were asked to watch a monitor screen displaying digits in a pseudo-random sequence in the center of the screen and to respond to any of three predetermined three-digit target sequences (2-4-6, 4-6-8 or 3-5-7) while ignoring distractors. The test measured reaction time to a specific stimulus in milliseconds [24].

**Figure 8:**
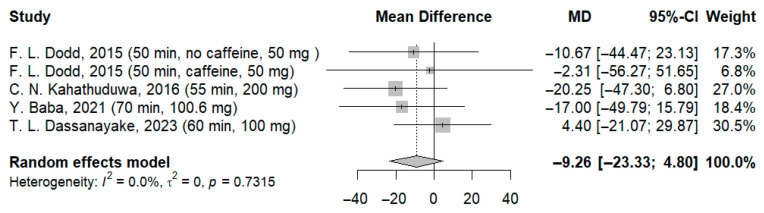
Forest plot of the time required to process visual stimuli I [21,22,23,24].

A total of four studies involving 125 participants were analyzed based on the results of the time required to process visual stimuli (RVIP—rapid visual information processing/RVRT—recognition visual reaction time/continuous performance). The meta-analysis revealed that the reaction time to visual stimuli improved compared to the control group, but not significantly (MD: −9.26 ms; 95%-CI [−23.33; 4.80]; I^2^ = 0.0%; *p* = 0.7315) (Figure 8).

**Figure 9:**
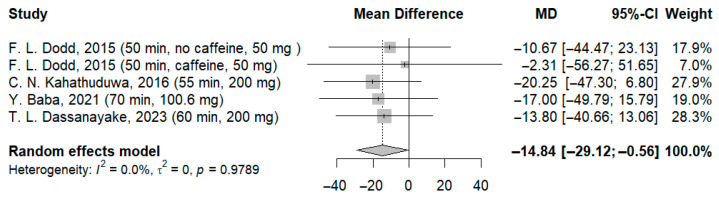
Forest plot of the time required to process visual stimuli II [21,22,23,24].

**Figure 10:**
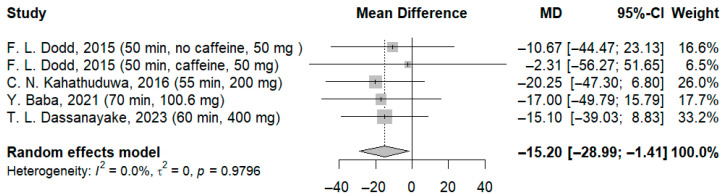
Forest plot of the time required to process visual stimuli III [21,22,23,24].

The use of higher doses resulted in a significant improvement in reaction time compared to the control group, with a dose-dependent manner (MD: −14.84 ms; 95%-CI [−29.12; −0.56]; I^2^ = 0.0%; *p* = 0.9789) (Figure 9), (MD: −15.20 ms; 95%-CI [−28.99; −1.41]; I^2^ = 0.0%; *p* = 0.9796) (Figure 10).

### 3.4. Assessment of Risk of Bias of the Included Studies and Publication Bias

**Figure 11:**
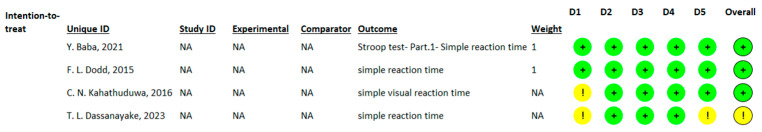
Risk of bias summary: review authors’ judgement about each risk of bias item for each included study—simple reaction time (some concerns (yellow) or low (green)) [21,22,23,24].

**Figure 12:**
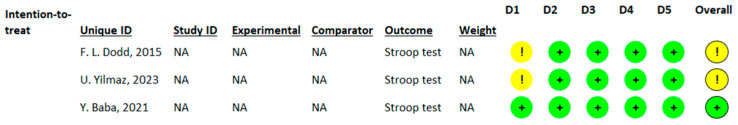
Risk of bias summary: review authors’ judgement about each risk of bias item for each included study—Stroop test (some concerns (yellow) or low (green)) [21,23,25].

**Figure 13:**
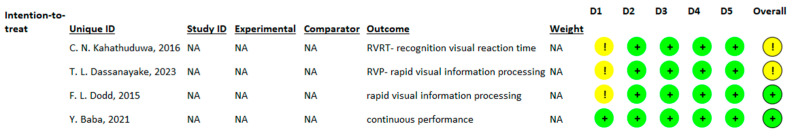
Risk of bias summary: review authors’ judgement about each risk of bias item for each included study—rapid visual information processing (some concerns (yellow) or low (green)) [21,22,23,24].

Each study was evaluated using the Cochrane Collaboration tool for assessing the risk of bias. For each domain, studies were judged to be at either high (red), unclear (yellow) or low (green) risk of bias; see Figure 11, Figure 12 and Figure 13. Two studies showed a low risk of randomization bias [21,23]. In the remaining studies, the method used for randomization was not mentioned; therefore, these studies were judged to have an unclear risk of selection bias. Studies which failed to describe the methods used for random sequence generation, allocation or blinding were reckoned to have an unclear risk of selection, performance and detection bias, respectively. No studies were judged as having a high risk of performance and detection bias due to the lack of blinding.

Potential sources of heterogeneity could have been determined by using subgroup and meta-regression analyses, but there were too few included studies to use these methods. The low number of the included studies did not allow for assessing the publication bias funnel plots.

### 3.5. Grade of Evidence

The GRADE assessment revealed that the certainty of evidence for l-theanine for cognitive enhancement in these settings was consistently low or very low, mainly due to the small sample sizes, inconsistency and imprecision (Table 5). Overall, while the acute administration of l-theanine may improve cognition, the confidence of these findings is limited.

### 3.6. Studies Included in the Qualitative Analysis

Three studies were excluded from the statistical analysis; however, the results of these may also contribute to the whole picture of the clinical efficacy of l-theanine. Haskell et al. conducted a controlled, randomized, double-blind, balanced crossover study to investigate the acute effects of l-theanine on cognition and mood in a total of 24 young healthy adults [26]. Simple reaction time and rapid visual information processing were measured pre- and post-treatment, 30 and 90 min after the administration of a 250 mL modified Peach Lite Lipton Iced Tea drink containing 250 mg of l-theanine or placebo according to a testing protocol. In the simple reaction time study, the participants were instructed to press the “Yes” response button as quickly as possible every time the word “Yes” was presented on the monitor. Reaction times were recorded in milliseconds (ms). In the rapid visual information processing study, the participant monitors a continuous series of digits for the targets of three consecutive odd or three consecutive even digits. The participants had to respond to the detection of a target string by pressing the “Yes” response button as quickly as possible [27]. The study measured reaction time in milliseconds and the accuracy of responses (%). The researchers noted that l-theanine alone had relatively little effect. The results of the use of l-theanine were not described in sufficient detail to include this trial in the meta-analysis. In this study, it was observed that the combination of l-theanine and caffeine resulted in improvements in both simple reaction time and the accuracy of rapid visual information processing [26].

Kahathuduwa et al. did not present all the results (i.e., baseline and endpoint reaction times) numerically in their publication from 2018; therefore, this article was excluded from the statistical analysis. The repeated measures, placebo-controlled, randomized 4-way crossover study by Kahathuduwa et al. aimed to assess the efficacy of 200 mg of l-theanine using the visual color stimulus discrimination reaction time task. Nine healthy adult men were included, and reaction time was measured 60 min after taking l-theanine. The reaction time of each target stimulus and the average reaction time to target stimuli within each functional run were displayed on the screen [28]. The study found that l-theanine and the combination of l-theanine and caffeine significantly improved reaction times for visual color discrimination by 27.8 milliseconds and 26.7 milliseconds, respectively, compared to the placebo [28].

Higashiyama et al. (2011) evaluated the effect of 200 mg l-theanine on attention and reaction time responses in a double-blind, repeated design study involving 18 healthy adult patients [29]. Before the study began, subjects were asked to complete the manifest anxiety scale (MAS), which was scored to place them in the high anxiety propensity or minimal anxiety propensity group, where the level of anxiety was directly proportional to the individual’s total anxiety score. Subjects in both groups were given either 200 mg of l-theanine solution or just a placebo, water, and then were administered the attention test or the reaction time response task at 15, 30, 45 and 60 min after the preparation was ingested and at baseline. In the attention task, subjects were asked to focus on a random number (1–9) on the screen and asked to press a button when the numbers 2, 5 or 8 appeared on the screen. In this study, the authors measured the number of times subjects answered correctly and the number of times they made a mistake, but the response time was not measured, so the study could not be included in the meta-analysis. In the other test of the study, participants were asked to react as quickly as they could to a 500 Hz frequency sound and to press the button and distinguish it from the distractor, a 1000 Hz frequency sound. The study found that subjects with a high anxiety propensity had significantly increased attention task scores between l-theanine and placebo treatments according to one-way ANOVA, and that changes in the reaction time response of the high anxiety propensity group compared to the baseline data showed a significant effect of l-theanine treatment compared to the placebo [29]. For simple reaction time studies, we included studies that measured reaction times to a visual stimulus, and this study measured reaction times to a sound stimulus; therefore, we did not consider it appropriate to include data from this study in the analysis.

## 4. Discussion

l-theanine is an unique naturally occurring amino acid that is synthesized primarily in the roots of plants through the enzymatic conversion of glutamate and ethylamine, catalyzed by theanine synthase [30]. Fresh tea leaves contain approximately 1–2% of l-theanine. Recently, l-theanine gained considerable attention as a food supplement. Several studies addressed the pharmacokinetics and pharmacodynamics of the molecule. The bioavailability of l-theanine is around 70%, and after ingestion it reaches its t_max_ within 0.8 h. Based on animal studies, l-theanine is safe. No toxicity was observed in rats given 2000 mg/kg for four weeks or 6500 mg/kg for two weeks. There was no evidence of carcinogenicity in rat models or genotoxicity in the Salmonella mutagenicity assay [31]. l-theanine crosses the blood–brain barrier via a leucine-preferred transport system and affects the central nervous system through several pathways, thereby exerting antidepressant and mood-enhancing effects [31,32,33]. L-theanin increased cognitive function in animal studies [34,35]. Mice with scopolamine-induced amnesia were treated with l-theanine (20 mg/kg, orally), and their cognitive decline was reversed through several mechanisms involving increased AMP-activated protein kinase, microtubule-associated protein 1 light chain 3-II (LC3-II) and beclin-1, and reduced phosphorylated protein kinase B (p-AKT) and mammalian target of rapamycin (p-mTOR) levels. Furthermore, l-theanine increased the expression of brain-derived neurotrophic factor (BDNF) and decreased caspase-3. l-theanine increased glutathione levels while decreasing malondialdehyde and tumor necrosis factor-alpha concentrations. l-theanine alleviated histopathological alterations, reduced amyloid-β accumulation and improved learning and memory performance in novel object recognition tests [34].

Several previous clinical trials and systematic reviews have aimed to assess the efficacy of l-theanine on cognition. However, based on our comprehensive literature review, the efficacy of l-theanine as monotherapy for any condition had not previously been investigated prior to our meta-analysis. Therefore, this meta-analysis aimed to summarize the available evidence on the efficacy of l-theanine in improving cognitive function.

In a previous meta-analysis, the efficacy of the combination of caffeine and l-theanine was examined. In the study of Camfield et al., the acute effects of tea constituents on cognition and mood were assessed. The applied products contained caffeine (40–250 mg), (-)-epigallocatechin gallate (135–300 mg) and l-theanine (12–250 mg). The authors investigated the acute effects of the products based on the Bond–Lader scale, the rapid visual information processing task, the attention switching task and the intersensory attention task 60–120 min after administration. Their meta-analysis revealed that the combination of caffeine and l-theanine increased attention switching accuracy and unisensory visual attention accuracy (first and second hours post-dose) compared to placebo and had a moderate effect on alertness one hour after administration and a small-to-moderate effect on alertness in the second hour [8].

A recent meta-analysis assessed the efficacy of l-theanine on sleep quality. The meta-analysis published by Bulman et al. assessed the effects of l-theanine based on 18 clinical trials involving 897 participants. Participants took 50 mg to 1000 mg of l-theanine daily for varying periods of time [36]. The results revealed that objective sleep measures did not improve; however, positive effects were observed on subjective measures of sleep onset latency, daytime dysfunction and overall sleep quality. However, a key limitation of this study was that it assessed different products containing l-theanine and other active ingredients.

To date, our study is the first meta-analysis to assess the efficacy of known doses of l-theanine for monotherapy on cognition. Based on a comprehensive literature search, five RCTs were identified, which included a total of 148 healthy adults. The effects of l-theanine on the outcomes tested in RCTs are summarized in Table 1, Table 2, Table 3 and Table 4. For the evaluation of the effects of a natural product, it is indispensably necessary to describe the applied product properly. In our meta-analysis, the effects of a well-described product were assessed. In the included trials, the posology of the study drug was well described, but the concomitant medications and caffeine consumption taken simultaneously with the study drug were not described in detail. Due to the limited number of included trials, it was not possible to properly assess publication bias using funnel plots. Based on the simple reaction time and Stoop test results, l-theanine in doses from 50 mg to approximately 500 mg did not affect cognitive function compared to a placebo. However, its effects on the third outcome, the processing of visual stimuli (RVIP—rapid visual information processing/RVRT—recognition visual reaction time/continuous performance), were significant in higher doses. The observed effects of l-theanine on cognition may be explained by previous studies in animals and humans, in which it was found to enhance autophagy, support neuronal survival, mitigate oxidative stress, modulate levels of gamma-aminobutyric acid (GABA) and inhibit glutamate receptors in the brain [16,34]. Yet our meta-analysis shows that the superiority of l-theanine over a placebo in enhancing cognition is not proven. Upon comparing our results with the literature data, the question arises as to whether l-theanine only affects cognition when combined with caffeine and whether this combination is more effective than caffeine alone. Our meta-analysis confirms that l-theanine is safe within the above dose range (i.e., 50–500 mg), as its side-effect profile did not differ from that of the placebo.

Compared to previous studies on this topic, the present meta-analysis has certain strengths. One of these is transparency: our meta-analysis was registered in the PROSPERO register, and we predefined a question to be answered, as well as the population, the comparator and the outcomes. No amendments were made to the information provided at registration. The systematic review and meta-analysis were conducted according to the original registered protocol, with no changes to the objectives, eligibility criteria, search strategy or methods. The use of explicit eligibility criteria resulted in the inclusion of all relevant articles on this topic. However, grey literature and unpublished studies were not systematically searched, potentially contributing to some publication bias.

Finally, due to resource constraints, the review did not include a formal statistical assessment of heterogeneity or sensitivity analyses, limiting the robustness of the conclusions. The major strength of this paper is that it only includes studies that assess known doses of l-theanine. Based on the available pharmacokinetic studies, the lag time for l-theanine (25–100 mg) from aqueous solutions is approximately 10 min, and the half-life is approximately 15–65 min. l-theanine reaches its maximum plasma concentration 30 min to two hours after oral administration [9]. In the studies included in our meta-analysis, the acute effects of l-theanine were measured 40–70 min after ingestion, which corresponds to the aforementioned t(max) times. Therefore, we can assume that the effects of l-theanine were measured at the optimal time to observe its maximum efficacy. The timing of the measurement might be correct, but the optimum doses of l-theanine are yet to be determined. In our meta-analysis, the doses varied from 50 to approx. 500 mg. Previous meta-analyses have observed the effects of l-theanine in combination with other tea constituents, such as caffeine. The l-theanine doses in these studies varied from 12 to 250 mg [8]. Products on the market containing l-theanine vary in content from 100 to 250 mg and are often recommended to aid sleep and improve cognition. Based on the results of our meta-analysis, when applied in higher doses, a significant improvement was observed in the processing of visual stimuli (RVIP—rapid visual information processing/RVRT—recognition visual reaction time). However, given the low certainty of the evidence, as assessed by GRADEs, these findings should be implemented with caution. The limitations of our literature review and meta-analysis are largely related to the low number of original studies. The effects of l-theanine were measured in various ways. This allowed us to analyze and compare the effects based on different tests, but it also leads to relatively small forest plots. And the number of participants involved in each study was also low; therefore, further, larger trials might change the estimate.

## 5. Conclusions

The beneficial effects of l-theanine on cognitive performance could not be confirmed by all test methods. It can be concluded that l-theanine has no significant effect on reaction time to a simple stimulus (MD: −0.46 ms; 95% [CI: −15.65; 14.73]), as its effects remained non-significant based on the Stroop test to both congruent (MD: −37.38 ms; 95%-CI [−86.39; 11.62]) and incongruent stimuli (MD: 109.28 ms; 95% CI [−8.72; 227.27]). However, a dose-dependent improvement was observed in RVIP (rapid visual information processing) and RVRT (recognition visual reaction time) (MD: −15.20 ms; 95%-CI [−28.99; −1.41]). The contradictory results could be explained by the fact that l-theanine only affects certain cognitive domains, but also by the low number of trials and the heterogeneity of the test preparations. Our meta-analysis partially supports the use of l-theanine as a monotherapy for cognitive enhancement, and it highlights the lack of clinical data concerning natural products. Further trials using standardized products with larger sample sizes are required to better assess efficacy.

## Figures and Tables

**Table 1:** Demographic characteristics of individuals in placebo-controlled randomized trials included in the quantitative analysis.

First Author, Year of Publication	Country	Study Type	Subgroup	Group	Sample Size	Patient Characteristics		References
						Age (Years; Mean)	No of Male Participants	
Dodd, 2015	UK	RCT	non-habitual caffeine consumers	l-theanine	12	20.4 (1.88)	5	[21]
				placebo	12			
			habitual caffeine consumers	l-theanine	12	23.3 (3.65)	5	
				placebo	12			
Kahathuduwa, 2016	Sri Lanka	RCT		l-theanine	20	21.9	20	[24]
				placebo	20			
Baba, 2021	Japan	RCT		l-theanine	26	57.7	12	[23]
				placebo	24	57.9	11	
Dassanayake, 2023	Sri Lanka	RCT		l-theanine	32	26.2 (2.2)	20	[24]
				placebo	32			
Yilmaz, 2023	Turkey	RCT		l-theanine	22	20.2	no information	[25]
				placebo	22			

**Table 2:** Characteristics of studies included in the final analysis of reaction time to simple stimulus detection (SRT).

First Author, Year of Publication	Country	Study Type	Subgroup	Group	Dose	Intervention Form	Sample Size (n)	Outcome	Results (Milisecundum)		Adverse Events	Reference
									Pre Dose	Post Dose		
Dodd, 2015	UK	RCT	non-habitual caffeine consumers	l-theanine	50 mg	capsule	12	Simple reaction time	329.85 ± 19.73 (mean ± SEM)	365.31 ± 20.98 (mean ± SEM)	No adverse events have been reported.	[21]
				placebo		capsule	12		326.54 ± 16.59	344.09 ± 15.26		
			habitual caffeine consumers	l-theanine	50 mg	capsule	12		288.71 ± 11.38	308.37 ± 11.77		
				placebo		capsule	12		294 ± 11.19	308.38 ± 11.85		
Kahathuduwa, 2016	Sri Lanka	RCT		l-theanine	200 mg	solution	20	Simple visual reaction time	230.30 (18.33) mean (SD)	235.40 (25.33) mean (SD)	None developed any adverse effects.	[22]
				placebo (distilled water)		liquid	20		224.20 (25.51)	223.65 (23.78)		
Baba, 2021	Japan	RCT		l-theanine	100.6 mg	capsule	26	(Stroop test: Part 1.) Simple reaction time	378 ± 138	337 ± 84	No adverse events have been reported.	[23]
				placebo (corn starch)		capsule	24		411 ± 145 mean ± SD	365 ± 62.0 mean ± SD		
Dassanayake, 2023 *	Sri Lanka	RCT		l-theanine	100 mg	solution	32	Simple reaction time	306.4 (45.0) mean (SD)	291.7 (40.6) mean (SD)	No adverse events have been reported.	[24]
				l-theanine	200 mg	solution	32		306.6 (53.4)	291.3 (44.6)		
				l-theanine	400 mg	solution	32		310.0 (51.9)	306.8 (51.5)		
				placebo (distilled water)		liquid	32		302.1 (52.1)	303.7 (43.7)		

* The data was collected/verified from the author.

**Table 3:** Characteristics of studies included in the final analysis of Stroop test.

First Author, Year of Publication	Country	Study Type	Subgroup	Group	Dose	Intervention Form	Sample Size (n)	Outcome	Results				Adverse Events	Reference
									Congruent		Incongruent			
									Pre Dose (ms)	Post Dose (ms)	Pre Dose (ms)	Post Dose (ms)		
Dodd, 2015	UK	RCT	non-habitual caffeine consumers	l-theanine	50 mg	capsule	12	Stroop test	692.9 ± 57.09	672.17 ± 40.69	Stroop interference RT (ms) 14.17 ± 14.36	Stroop interference RT (ms) 17.67 ± 4.46	No adverse events have been reported.	[21]
				placebo		capsule	12		628.92 ± 26.50	660.33 ± 28.40	Stroop interference RT (ms) 12.67 ± 12.96	Stroop interference RT (ms) 7.92 ± 8.6		
			habitual caffeine consumers	l-theanine	50 mg	capsule	12		659.5 ± 49.91	637.08 ± 25.4	Stroop interference RT (ms) 25.00 ± 15.19	Stroop interference RT (ms) 43.92 ± 9.26		
				placebo		capsule	12		651.67 ± 26.36	641.25 ± 22.65	Stroop interference RT (ms) 11.33 ± 15.55	Stroop interference RT (ms) 32.92 ± 7.86		
Yilmaz, 2023	Turkey	RCT		l-theanine	6 mg/kg	powder	22	Stroop test		616 ± 108.95		902.5 ± 144.05	No adverse events have been reported.	[25]
				placebo (maltodextrin)		powder	22			720 * ± 129.18		1025 ± 122.24		
Baba, 2021	Japan	RCT		l-theanine	100.6 mg	capsule	26	Stroop test	706 ± 122	667 ± 97.8	802 ± 121	761 ± 120	No adverse events have been reported.	[23]
				placebo (corn starch)		capsule	24		734 ± 124	706 ± 133	841 ± 129	809 ± 98.2		

* The data was collected/verified from the author.

**Table 4:** Characteristics of studies included in the analysis of time required to process visual stimuli (RVIP—rapid visual information processing/RVRT—recognition visual reaction time/continuous performance).

First Author, Year of Publication	Country	Study Type	Subgroup	Group	Dose	Intervention Form	Sample Size	Outcome	Results (Millisecond)		Adverse Events	Reference
									Pre dose	Post dose		
Dodd, 2015	UK	RCT	non-habitual caffeine consumers	l-theanine	50 mg	capsule	11	Rapid visual information processing	480.70 ± 13.99 mean ± SEM	471.87 ± 13.28 mean ± SEM	No adverse events have been reported.	[21]
				placebo		capsule	11		481.36 ± 15.76	482.54 ± 11.00		
			habitual caffeine consumers	l-theanine	50 mg	capsule	12		501.75 ± 18.82	511.05 ± 17.77		
				placebo		capsule	12		502.55 ± 18.02	513.36 ± 21.03		
Kahathuduwa, 2016	Sri Lanka	RCT		l-theanine	200 mg	solution	20	Recognition visual reaction time	385.60 (54.27) mean (SD)	363.20 (42.85) mean (SD)	None developed any adverse effects.	[22]
				placebo (distilled water)		liquid	20		380.55 (50.28)	383.45 (44.43)		
Baba, 2021	Japan	RCT		l-theanine	100.6 mg	capsule	26	Time required to process visual stimuli–Cognitrax subtest–continuous performance	492 ± 80.4 mean ± SD	483 ± 72.1 mean ± SD	No adverse events have been reported.	[23]
				placebo (corn starch)		capsule	24		511 ± 50.7	500 ± 43.8		
Dassanayake, 2023 *	Sri Lanka	RCT		l-theanine	100 mg	solution	32	Rapid visual information processing	382.5 (50.8) mean (SD)	381.3 (50.0) mean (SD)	No adverse events have been reported.	[24]
				l-theanine	200 mg	solution	32		380.8 (58.1)	363.1 (55.7)		
				l-theanine	400 mg	solution	32		375.8 (48.6)	361.8 (43.2)		
				placebo (distilled water)		liquid	32		383.5 (46.5)	376.9 (53.9)		

* The data was collected/verified from the author.

**Table 5:** Summary for quality of evidence; GRADE assessment.

Certainty Assessment							Number of Patients		Effect		Certainty	Importance
Number of Studies ^a^	Study Design	Risk of Bias	Inconsistency	Indirectness	Imprecision	Other Considerations	L-Theanine ^c^	Placebo ^c^	Relative (95% CI)	Absolute (95% CI)		
Effects of l-theanine on reaction time to simple stimulus												
5	RCT	Not serious	Serious	No -serious	Not serious	None	102 ^b^	100 ^b^	-	MD: −0.46 ms (CI: −15.65; 14.73) ^c^	⨁⨁◯◯ Low ^d,e^	Critical
Effects of l-theanine on congruent stimuli based on the Stroop test												
4	RCT	Not serious	Serious	Not serious	Serious	None	72	70	-	MD: −37.38 ms (CI −86.39; 11.62)	⨁⨁◯◯ Low ^d,e^	Critical
Effects of l-theanine on incongruent stimuli based on the Stroop test												
4	RCT	Not serious	Very serious	Not serious	Very serious	None	72	70	-	MD: 109.28 ms (CI −8.72; 227.27)	⨁◯◯◯ Very low ^d,e^	Critical
Effects of l-theanine on time required to process visual stimuli												
5	RCT	Not serious	Not serious	Not serious	Not serious	None	101	99	-	MD: −15.20 ms (CI −28.99; −1.41) ^f^	⨁⨁◯◯ Low ^d,e^	Critical

Abbreviations: MD: mean difference; CI: 95% confidence interval; RCT: randomized controlled trial. Explanations: ^a^: Two different l-theanine arms were used in the plots from the study by Dodd et al., so here we count this study as two. ^b^: It can be considered serious based on two of the plots. ^c^: When using 200 mg l-theanine in the study by Dassanayake et al. ^d^: A low patient number in all the articles. ^e^: No information on habitual caffeine intake in some of the studies. ^f^: When using 400 mg l-theanine in the study by Dassanayake et al.

## Supplementary Materials

The following supporting information can be downloaded at https://www.mdpi.com/article/10.3390/jcm14217710/s1, Table S1: List of excluded studies and exclusion reason [1,2,11,13,14,15,37,38,39,40,41,42,43,44,45,46,47,48,49,50,51,52,53,54,55,56,57,58,59,60,61,62,63,64,65,66,67,68,69,70,71,72,73,74,75,76,77,78,79,80,81,82,83,84,85,86,87,88,89,90,91,92].
