# Supplementary material for: Promising, but Not Completely Conclusive—The Effect of l-Theanine on Cognitive Performance Based on the Systematic Review and Meta-Analysis of Randomized Placebo-Controlled Clinical Trials
Source: J Clin Med. 2025 Oct 30;14(21):7710. doi: 10.3390/jcm14217710 (PMC12609247; doi:10.3390/jcm14217710)
Supplement: Supplementary file 1 [file jcm-14-07710-s001.zip › jcm-3939052-supplementary.pdf]

Supplementary Material

Promising, but Not Completely Conclusive—The Effect of L-Theanine on Cognitive Performance Based on the Systematic Review and Meta-Analysis of Randomized Placebo-Controlled Clinical Trials

Rebeka Olga Mátyus [1], Zsóka Szikora [1], Diána Bodó [1], Bettina Vargáné Szabó [1], Éva Csupor [2], Dezső Csupor [1,*] and Barbara Tóth [1,*]

Affiliations

[1] Faculty of Pharmacy, Institute of Clinical Pharmacy, University of Szeged, 6725 Szeged, Hungary; matyus.rebeka.olga@med.u-szeged.hu or matyus.rebeka@szte.hu (R.O.M.); szikora.zsoka@szte.hu (Z.S.); vargane.szabo.bettina@szte.hu (B.V.S.)

[2] Department of Pediatrics and Pediatric Health Center, Albert Szent-Györgyi Health Centre, 6725 Szeged, Hungary

[*] Correspondence: csupor.dezso@szte.hu (D.C.); toth.barbara.eva@szte.hu (B.T.);
Tel.: +36-62-544-921 (D.C.)

Table S1 List of excluded studies and exclusion reason

Primary exclusion reason	Reference number
Full text article not available, abstract	[1] – [6]
Not a human clinical study or not a double blind randomised controlled trial with L-theanine	[7] – [19]
Does not correspond to the PICO requirements	[20] – [63]

1. Weerasinghe VS, Kahathuduwa C, Amarakoon T, Dassanayake T. Synergistic effect of theanine and caffeine on visual reaction time, evoked potentials and cognitive event related potentials. J Clin Neurophysiol. 2015;32(4):396.

2. Dodd FL, Kennedy DO, Riby LM, Wilde A, Haskell CF. An evaluation of the cerebral blood flow, cognitive and mood effects of caffeine and l-theanine both alone and in combination. Appetite. 2011;57(2):557.

3. Yoneda Y. Green tea amino acid products for prevention of cognitive declines. JOURNAL OF PHARMACOLOGICAL SCIENCES. 2017;133(3, S):S22.

4. Yoneda Y, Kuramoto N. The green tea amino acid theanine for possible improvement of cognitive declines. JOURNAL OF NEUROCHEMISTRY. 2017;142(1, SI):144.

5. Eri W, Kuchta K, Mari K, Rauwald HW, Kamei T, Imanishi J. On the fatigue reducing effects of Japanese green tea (Camellia sinensis (L.) Kuntze) from Uji. Planta Med [Internet]. 2013;79(13). Elérhető: https://www.embase.com/search/results?subaction=viewrecord&id=L71353926&from=export

6. Kovacs EMR, Balentine DA, Einöther SJL, Rycroft JA, De Bruin EA. Emerging science demonstrates that L-theanine and caffeine in combination can help improve attention. FASEB J [Internet]. 2009;23(S1). Elérhető: https://www.embase.com/search/results?subaction=viewrecord&id=L70055894&from=export

7. Miller LA. A cup of tea. J Perinat Neonatal Nurs. 2010;24(3):284–5.

8. Natarajan R. A story in a tea cup. BMJ (Online) [Internet]. 2017;356((Natarajan R.) Medical Research Foundation, Nungambakkam Chennai, India). Elérhető: https://www.embase.com/search/results?subaction=viewrecord&id=L614095273&from=export

9. Mason R. 200 mg of Zen: L-Theanine boosts alpha waves, promotes alert relaxation. Altern Complement Ther. 2001;7(2):91–5.

10. Shirai Y, Kuriki K, Otsuka R, Kato Y, Nishita Y, Tange C, és mtsai. Association between green tea intake and risk of cognitive decline, considering glycated hemoglobin level, in older Japanese adults: the NILS-LSA study. NAGOYA JOURNAL OF MEDICAL SCIENCE. 2019;81(4):655–66.

11. Noguchi-Shinohara M, Yuki S, Dohmoto C, Ikeda Y, Samuraki M, Iwasa K, és mtsai. Consumption of green tea, but not black tea or coffee, is associated with reduced risk of cognitive decline. PLoS ONE [Internet]. 2014;9(5). Elérhető: https://www.embase.com/search/results?subaction=viewrecord&id=L373152961&from=export

12. Kuriyama S, Hozawa A, Ohmori K, Shimazu T, Matsui T, Ebihara S, és mtsai. Green tea consumption and cognitive function: A cross-sectional study from the Tsurugaya Project. Am J Clin Nutr. 2006;83(2):355–61.

13. Ross SM. L-theanine (suntheanin): effects of L-theanine, an amino acid derived from Camellia sinensis (green tea), on stress response parameters. Holist Nurs Pract. 2014;28(1):65–8.

14. Kawada T. Tea and coffee consumption, cognitive impairment and prognosis in older inhabitants. EXCLI J. 2020;19((Kawada T., kawada@nms.ac.jp) Department of Hygiene and Public Health, Nippon Medical School, 1-1-5 Sendagi, Bunkyo-Ku, Tokyo, Japan):1385–6.

15. Feng L, Yan Z, Sun B, Cai C, Jiang H, Kua EH, és mtsai. Tea Consumption and Depressive Symptoms in Older People in Rural China. JOURNAL OF THE AMERICAN GERIATRICS SOCIETY. 2013;61(11):1943–7.

16. Williamson C. Tea drinking prevents memory loss? Nutr Bull. 2005;30(1):2–5.

17. McAllister MJ, Martaindale MH, Dillard CC, McCullough R. Impact of L-theanine and L-tyrosine on markers of stress and cognitive performance in response to a virtual reality based active shooter training drill. Stress [Internet]. 2024;27(1). Elérhető: https://www.embase.com/search/results?subaction=viewrecord&id=L2030506600&from=export

18. Füeßl HS. Green tea consumption promotes the cognitive function. MMW-Fortschr Med. 2006;148(27–28):19.

19. Flores MF, Martins A, Schimidt HL, Santos FW, Izquierdo I, Mello-Carpes PB, és mtsai. Effects of green tea and physical exercise on memory impairments associated with aging. NEUROCHEMISTRY INTERNATIONAL. 2014;78:53–60.

20. Park SK, Jung IC, Lee WK, Lee YS, Park HK, Go HJ, és mtsai. A combination of green tea extract and l-theanine improves memory and attention in subjects with mild cognitive impairment: A double-blind placebo-controlled study. J Med Food. 2011;14(4):334–43.

21. Furushima D, Sugiyama I, Nomura Y, Unno K, Yamada H. Effect of Combined Ingestion of l-Theanine and l-Arginine for Short-Term Psychological Stress in Young Adults: A Randomized Placebo-Controlled Study. J Nutr Sci Vitaminol. 2022;68(6):540–6.

22. Einöther SJL, Martens VEG, Rycroft JA, De Bruin EA. l-Theanine and caffeine improve task switching but not intersensory attention or subjective alertness. Appetite. 2010;54(2):406–9.

23. Golden E, Johnson M, Jones M, Viglizzo R, Bobe J, Zimmerman N. Measuring the Effects of Caffeine and L-Theanine on Cognitive Performance: A Protocol for Self-Directed, Mobile N-of-1 Studies. FRONTIERS IN COMPUTER SCIENCE. 2020. február 13.;2.

24. Giesbrecht T, Rycroft JA, Rowson MJ, De Bruin EA. The combination of L-theanine and caffeine improves cognitive performance and increases subjective alertness. Nutr Neurosci. 2010;13(6):283–90.

25. Owen GN, Parnell H, De Bruin EA, Rycroft JA. The combined effects of L-theanine and caffeine on cognitive performance and mood. Nutr Neurosci. 2008;11(4):193–9.

26. Scholey A, Burns A, Pase M, Pipingas A. Acute cognitive, mood and cardiovascular effects of green and black tea. Proc Nutr Soc [Internet]. 2020;79(OCE2). Elérhető: https://www.embase.com/search/results?subaction=viewrecord&id=L633556142&from=export

27. Medrano M, Molina-Hidalgo C, Alcantara JMA, Ruiz JR, Jurado-Fasoli L. Acute Effect of a Dietary Multi-Ingredient Nootropic as a Cognitive Enhancer in Young Healthy Adults: A Randomized, Triple-Blinded, Placebo-Controlled, Crossover Trial. FRONTIERS IN NUTRITION. 2022. május 12.;9.

28. Unno K, Yamada H, Iguchi K, Ishida H, Iwao Y, Morita A, és mtsai. Anti-stress effect of green tea with lowered caffeine on humans: A pilot study. Biol Pharm Bull. 2017;40(6):902–9.

29. White DJ, de Klerk S, Woods W, Gondalia S, Noonan C, Scholey AB. Anti-stress, behavioural and magnetoencephalography effects of an l-theanine-based nutrient drink: A randomised, double-blind, placebo-controlled, crossover trial. Nutrients [Internet]. 2016;8(1). Elérhető: https://www.embase.com/search/results?subaction=viewrecord&id=L607786317&from=export

30. Zhang T, Li L, Liu Y, Zhong D, Tao Y, Jiang X, és mtsai. Effect of coffee and green tea on executive ability and plasma levels of inflammatory factors in soldiers with 48-hour total sleep deprivation. Int J Clin Exp Med. 2016;9(10):19354–62.

31. Wang H, Sun W, Chang Y, Wu Z, Xu Y, Wang E, és mtsai. Effect of green tea consumption on human brain function in resting-state functional MRI. Asia Pac J Clin Nutr. 2019;28(4):740–6.

32. Zhang Q, Yang H, Wang J, Li A, Zhang W, Cui X, és mtsai. Effect of green tea on reward learning in healthy individuals: A randomized, double-blind, placebo-controlled pilot study. Nutr J [Internet]. 2013;12(1). Elérhető: https://www.embase.com/search/results?subaction=viewrecord&id=L52642935&from=export

33. Okello EJ, Abadi AM, Abadi SA. Effects of green and black tea consumption on brain wave activities in healthy volunteers as measured by a simplified Electroencephalogram (EEG): A feasibility study. Nutr Neurosci. 2016;19(5):196–205.

34. Sakurai K, Shen C, Ezaki Y, Inamura N, Fukushima Y, Masuoka N, és mtsai. Effects of matcha green tea powder on cognitive functions of community-dwelling elderly individuals. Nutrients. 2020;12(12):1–15.

35. Shirai N, Higuchi T, Suzuki H. Effects of Simultaneous Intake of Green Tea Extracts and Fish Oil on Cognitive Function and Plasma Lipids in the Elderly. JOURNAL OF THE JAPANESE SOCIETY FOR FOOD SCIENCE AND TECHNOLOGY-NIPPON SHOKUHIN KAGAKU KOGAKU KAISHI. 2015;62(2):88–94.

36. Schmidt A, Hammann F, Wölnerhanssen B, Meyer-Gerspach AC, Drewe J, Beglinger C, és mtsai. Green tea extract enhances parieto-frontal connectivity during working memory processing. Psychopharmacology. 2014;231(19):3879–88.

37. Ide K, Yamada H, Takuma N, Park M, Wakamiya N, Nakase J, és mtsai. Green tea consumption affects cognitive dysfunction in the elderly: A pilot study. Nutrients. 2014;6(10):4032–42.

38. Rajagoplan UM. Green tea could improve the performance of cognitive tasks: a pilot study with wearable brain imaging device. In: 2018 INTERNATIONAL CONFERENCE ON ADVANCED MECHATRONIC SYSTEMS (ICAMECHS). Int Journal Modeling, Identificat & Control; IEEE; ZHENGZHOU Univ Aeronaut; Xinlan Coll Henan Normal Univ; Int Journal Adv Mechatronic Systems; 2018. o. 178–83. (International Conference on Advanced Mechatronic Systems).

39. Gibson N, Baker D, Sharples A, Braakhuis A. Improving Mental Performance in an Athletic Population with the Use of Ārepa(®), a Blackcurrant Based Nootropic Drink: A Randomized Control Trial. Antioxidants (Basel). 2020. április 15.;9(4).

40. Yoto A, Murao S, Nakamura Y, Yokogoshi H. Intake of green tea inhibited increase of salivary chromogranin A after mental task stress loads. J Physiol Anthropol. 2014;33((Yoto A., ai_yoto@hotmail.com; Murao S.; Nakamura Y.; Yokogoshi H.) School of Food and Nutritional Sciences, University of Shizuoka, Shizuoka, Japan.):20.

41. Borgwardt S, Hammann F, Scheffler K, Kreuter M, Drewe J, Beglinger C. Neural effects of green tea extract on dorsolateral prefrontal cortex. Eur J Clin Nutr. 2012;66(11):1187–92.

42. Small BJ, Rawson KS, Martin C, Eisel SL, Sanberg CD, McEvoy CL, és mtsai. Nutraceutical intervention improves older adults’ cognitive functioning. Rejuvenation Res. 2014;17(1):27–32.

43. Williams J, D’Cunha NM, Kellett J, Georgousopoulou EN, McKune AJ, Mellor DD, és mtsai. Physicochemical, antioxidant and sensory properties of Mango Sorbet containing L-theanine as a potential functional food product. J Food Sci Technol. 2022. december;59(12):4833–43.

44. Unno K, Noda S, Kawasaki Y, Yamada H, Morita A, Iguchi K, és mtsai. Reduced Stress and Improved Sleep Quality Caused by Green Tea Are Associated with a Reduced Caffeine Content. NUTRIENTS. 2017;9(7).

45. Bryan J, Tuckey M, Einöther SJL, Garczarek U, Garrick A, De Bruin EA. Relationships between tea and other beverage consumption to work performance and mood. Appetite. 2012;58(1):339–46.

46. Cicero AF, Bove M, Colletti A, Rizzo M, Fogacci F, Giovannini M, és mtsai. Short-Term Impact of a Combined Nutraceutical on Cognitive Function, Perceived Stress and Depression in Young Elderly with Cognitive Impairment: A Pilot, Double-Blind, Randomized Clinical Trial. J Prev Alzheimers Dis. 2017;4(1):12–5.

47. Unno K, Furushima D, Hamamoto S, Iguchi K, Yamada H, Morita A, és mtsai. Stress-reducing function of matcha green tea in animal experiments and clinical trials. Nutrients [Internet]. 2018;10(10). Elérhető: https://www.embase.com/search/results?subaction=viewrecord&id=L624498184&from=export

48. Williams J, McKune AJ, Georgousopoulou EN, Kellett J, D'Cunha NM, Sergi D, és mtsai. The Effect of L-Theanine Incorporated in a Functional Food Product (Mango Sorbet) on Physiological Responses in Healthy Males: A Pilot Randomised Controlled Trial. FOODS. 2020;9(3).

49. Unno K, Tanida N, Ishii N, Yamamoto H, Iguchi K, Hoshino M, és mtsai. Anti-stress effect of theanine on students during pharmacy practice: Positive correlation among salivary α-amylase activity, trait anxiety and subjective stress. PHARMACOLOGY BIOCHEMISTRY AND BEHAVIOR. 2013;111:128–35.

50. Hidese S, Ota M, Wakabayashi C, Noda T, Ozawa H, Okubo T, és mtsai. Effects of chronic l-theanine administration in patients with major depressive disorder: An open-label study. Acta Neuropsychiatr. 2017;29(2):72–9.

51. Yoto A, Motoki M, Murao S, Yokogoshi H. Effects of L-theanine or caffeine intake on changes in blood pressure under physical and psychological stresses. J Physiol Anthropol. 2012;31((Yoto A.) Laboratory of Nutritional Biochemistry, School of Food and Nutritional Sciences, University of Shizuoka, 52-1 Yada, Suruga-ku, Shizuoka 422-8526, Japan.):28.

52. Nobre AC, Rao A, Owen GN. L-theanine, a natural constituent in tea, and its effect on mental state. Asia Pac J Clin Nutr. 2008;17 Suppl 1((Nobre A.C.; Rao A.; Owen G.N.) Unilever Food and Health Research Institute, Olivier van Noortlaan 120, Postbus 114, 3130 AC Vlaardingen, The Netherlands.):167–8.

53. Kimura K, Ozeki M, Juneja LR, Ohira H. l-Theanine reduces psychological and physiological stress responses. Biol Psychol. 2007;74(1):39–45.

54. Foxe JJ, Morie KP, Laud PJ, Rowson MJ, de Bruin EA, Kelly SP. Assessing the effects of caffeine and theanine on the maintenance of vigilance during a sustained attention task. Neuropharmacology. 2012;62(7):2320–7.

55. Giles GE, Mahoney CR, Brunyé TT, Taylor HA, Kanarek RB. Caffeine and theanine exert opposite effects on attention under emotional arousal. Can J Physiol Pharmacol. 2017;95(1):93–100.

56. Kelly SP, Gomez-Ramirez M, Montesi JL, Foxe JJ. L-theanine and caffeine in combination affect human cognition as evidenced by oscillatory alpha-band activity and attention task performance. J Nutr. 2008;138(8):1572S-1577S.

57. Rogers PJ, Smith JE, Heatherley SV, Pleydell-Pearce CW. Time for tea: Mood, blood pressure and cognitive performance effects of caffeine and theanine administered alone and together. Psychopharmacology. 2008;195(4):569–77.

58. Gomez-Ramirez M, Kelly SP, Montesi JL, Foxe JJ. The effects of l-theanine on alpha-band oscillatory brain activity during a visuo-spatial attention task. Brain Topogr. 2009;22(1):44–51.

59. Ide K, Wakamiya N, Park M, Takuma N, Fujii S, Nakahara A, és mtsai. Effects of green tea consumption on cognitive dysfunction: An exploratory clinical study. J Neurol Sci. 2013;333((Ide K.; Wakamiya N.; Park M.; Yamada H.) School of Pharmaceutical Sciences, University of Shizuoka, Shizuoka, Japan):e298.

60. Ide K, Yamada H, Takuma N, Kawasaki Y, Harada S, Nakase J, és mtsai. Effects of green tea consumption on cognitive dysfunction in an elderly population: A randomized placebo-controlled study. Nutr J [Internet]. 2016;15(1). Elérhető: https://www.embase.com/search/results?subaction=viewrecord&id=L611093033&from=export

61. Sarris J, Byrne GJ, Cribb L, Oliver G, Murphy J, Macdonald P, és mtsai. L-theanine in the adjunctive treatment of generalized anxiety disorder: A double-blind, randomised, placebo-controlled trial. J Psychiatr Res. 2019;110((Sarris J., j.sarris@westernsydney.edu.au; Karamacoska D.; Ee C.; Birling Y.) NICM Health Research Institute, Western Sydney University, Westmead, NSW, Australia):31–7.

62. Lyon MR, Kapoor MP, Juneja LR. The Effects of L-Theanine (Suntheanine®) on Objective Sleep Quality in Boys with Attention Deficit Hyperactivity Disorder (ADHD): a Randomized, Double-blind, Placebo-controlled Clinical Trial. ALTERNATIVE MEDICINE REVIEW. 2011;16(4):348–54.

63. Hidese S, Ogawa S, Ota M, Ishida I, Yasukawa Z, Ozeki M, és mtsai. Effects of L-Theanine Administration on Stress-Related Symptoms and Cognitive Functions in Healthy Adults: A Randomized Controlled Trial. Nutrients [Internet]. 2019;11(10). Elérhető: https://www.embase.com/search/results?subaction=viewrecord&id=L2002736623&from=export
